# Find your village: co-designing a community asset-based peer support intervention for wellbeing, social connectedness, and early child development among migrant families during the first 1001 days

**DOI:** 10.1080/16549716.2026.2731693

**Published:** 2026-09-21

**Authors:** Tom Allport, Samira Musse, Jenny Ingram, Gene Feder, Fatumo Osman, Sabi Redwood

**Affiliations:** a Centre for Academic Child Health, Bristol Medical School, University of Bristol, Bristol, UK; b Community Children’s Health Partnership, Sirona Care & Health, Bristol, UK; c Executive and Operations, Barton Hill Activity Club, Bristol, UK; d Centre for Academic Primary Care, Bristol Medical School, University of Bristol, Bristol, UK; e School of Health and Welfare, Dalarna University, Falun, Sweden; f National Institute of Health Research Applied Research Collaborative for the West of England (NIHR ARC-West), Bristol, UK

**Keywords:** Migration, early child development, wellbeing, peer support, participatory mixed-methods complex intervention study

## Abstract

**Background:**

One in five children born in England and Wales has a parent born in the Global South. Migrant families are often marginalised, experience adverse environments, lack social support, and have highly stressed pregnancies; their children are at risk of adverse long-term outcomes. Interventions to improve early child development are highly effective and cost-effective, but often do not reach marginalised communities effectively.

**Objectives:**

To develop an asset-based community peer support intervention to improve family wellbeing, social connectedness, and early child development, addressing one migrant community’s strengths and needs in detail; and to articulate its transferability to different contexts.

**Methods:**

This participatory mixed-methods approach to complex intervention design was co-produced with community partners. We constructed an ecocultural model of challenges to early child development in local Somali refugee families, integrating findings from public and professional consultations; systematic reviews of qualitative and quantitative evidence; and local qualitative research and epidemiology. We then collaboratively developed, refined, and prototyped a flexible asset-based peer support intervention to address potentially modifiable factors.

**Results:**

The key transferable functions of this intervention are: reaching out to engage, enable, and advocate for families; organising group activities; and advocating for neighbourhood environment improvements. This has potential to improve wellbeing, child development, social connectedness, and relationships with agencies.

**Conclusions:**

This intervention is designed to mobilise migrant community assets, and improve wellbeing, developmental opportunities, and community connectedness, flexibly and transferably through local co-production. It requires evaluation for its effectiveness and cost-effectiveness, and for potential to reduce inequalities and disrupt cycles of disadvantage.

## Paper Context

- **Main findings**: A co-produced complex intervention design process produced a flexible asset-based peer support intervention in pregnancy and the first 2 years of life (the first 1001 days) for communities with migrant heritage.
- **Added knowledge**: This peer support intervention addresses potentially modifiable factors within the complex context of challenges to early child development for disadvantaged and marginalised migrant families in the Global North.
- **Global health impact for policy and action**: This flexible, transferable approach to co-producing these asset-based peer support interventions has potential to be delivered cost-effectively on a large scale, adapting to local strengths and needs, to reduce inequalities in experience and outcome for children and families with migrant heritage in the Global North.

## Background

Rates of international migration continue to increase [1]. In recent years, 22–24% of children born in England and Wales had a parent born in the Global South [2]. Migration to the Global North can bring many challenges for families, especially coming from cultures where ‘it takes a village to raise a child’ [3,4]. Migrant women are at high risk of adverse experience and outcome of pregnancy and childbirth, with care obstructed by structural, organisational, social, personal, and cultural barriers [5–9]. Parents who feel stressed/distressed, isolated, and marginalised can experience many difficulties bringing up children in neighbourhoods lacking in resources, which combine to interfere with how children and young people socialise, learn and mature [10–15]. These challenges present pressing concerns about social-emotional development for children with migrant heritage [16–18].

In contrast, strong and sustainable communities build wellbeing, security, and social connections, which improve people’s life chances [19,20]. Promoting social connectedness and enhancing support has wide value [21], but may be especially important for migrants and families from communal or collective child-rearing cultures [3,22–24]. Young children need opportunities for play and social interaction to develop and socialise confidently [25–27]. Social support for pregnant women in diverse and migrant communities is linked with their children’s social-emotional development [28]. Social support and connectedness therefore represent a key focus for advocacy and intervention [29,30].

Effective inequality-reducing public health action, coordinating local community, voluntary and statutory action has important potential to reduce inequalities in wellbeing, child development and longer-term outcomes [31,32]. Interventions to improve early child development are highly effective and cost-effective, especially if they operate from pregnancy onwards (‘the first 1001 days of life’) [33]. The evidence of long‑term benefits and cost savings from UK government Sure Start early years initiatives started in the late 1990s (https://www.daynurseries.co.uk/advice/what-is-a-sure-start-childrens-centre) are continuing to accumulate [34]. Many factors may challenge how accessible and helpful health services are for communities with migrant heritage [35]. Their uptake of statutory preventive services in pregnancy and the early years is often low [36]. Intervention reviews and meta-analyses underline the effectiveness of peer support [37–39], including with migrants [40], especially when they build trust [41], and recommend multi-faceted, asset- and group-based, proportionate universalist approaches [31].

In this paper, therefore, we report our innovative approach to developing asset-based community peer support interventions to improve wellbeing and early child development, working from local exemplars in Bristol, UK, designed to be transferable to other local contexts of migration and marginalisation. This is an intervention-development and theory-informed design study, with outcomes presented as anticipated or hypothesised.

## Methods

### Participatory mixed-methods approach to complex intervention design

**Figure 1.**
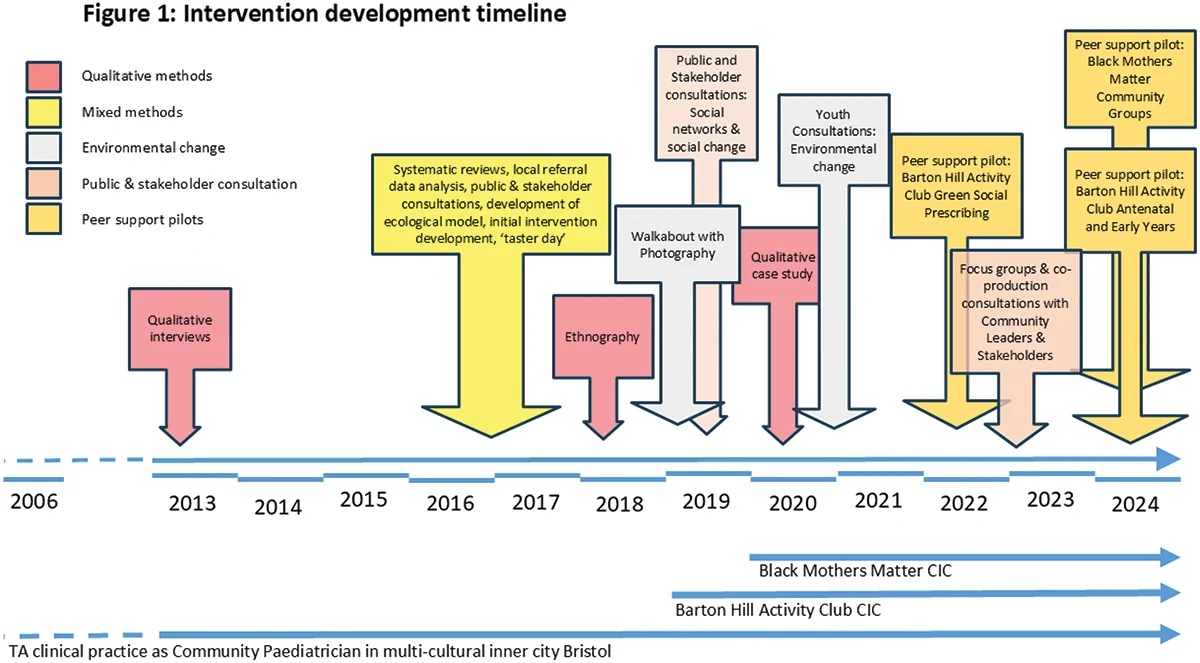
Intervention development timeline.

We structured this intervention development process according to revised MRC guidance for complex interventions [42], and the framework described by Hawkins et al [43]. We have followed TIDieR-PHP reporting guidelines [44], to describe processes of evidence review and stakeholder consultation, co-production, intervention design, and prototyping. The timeline for intervention development is portrayed in Figure 1. This was an iterative, co-produced process, incorporating a number of individual studies presented elsewhere [3,13,18,28,45–52]. The many public involvement and co-production meetings underpinning this report drew on 10 years of partnership working. They were not recorded, transcribed, or analysed as formal qualitative research; instead, they provided a rich, ongoing process for developing shared understanding of the relevant context, mechanisms, intervention potential, and practical steps for piloting and evaluation.

### Evidence review and stakeholder consultation (2016–17)

Our mixed methods evidence review included qualitative interviews [3], collecting local referral data [18], systematic quantitative and qualitative evidence reviews [48,49].

Our starting point for community engagement was with the local Somali community, seeking to develop local, culturally informed and relevant interventions [53–55]. Somali people are one of the world’s largest diasporas, estimated at over 1.9 million [1], with forced migration from conflict and complex transnational networks [56]. The UK has the largest and longest-established Somali community in Europe [57]; in Bristol 5% of children (about 4000) are of Somali origin.

We undertook public involvement meetings (3 meetings, each 1–1_1/2_h) involving 20 participants, 14 women/6 men, with Somali community members, with interpretation where required. Community members (local parents of children under 5 with Somali heritage) were identified through Bristol Somali Resource Centre and the outreach team of a local Nursery/Children’s Centre. We also held professional group consultations (each 1 h, *n* = 3, involving respectively Health Visitors, Speech & Language Therapists, and Early Years educational professionals), involving a total of 30 professionals, identified through local NHS and Education networks. Discussion in each meeting was semi-structured around themes of strengths, needs, barriers, and potential for change.

### Development of ecocultural model (2016–17)

We drew on the above data, together with further informal discussion within established local relationships, and our own experience, to construct an ecocultural model of challenges to early child development in Somali migrants to the UK. Factors (contexts, processes, experience, demands, resources) considered relevant by participants to understand how the context might challenge children’s developmental progress, together with those arising from systematic reviews, were assembled and ordered according to their levels of operation within the macro-, exo- and meso- and micro-systems [58–60]. The intention, following ecocultural thinking [55], was to understand the factors systemically or interdependently, considering both activities and experience [61]. The model was discussed with community partners and in further consultations with community group leaders, and feedback incorporated in the model presented in Figure 1.

### Asset-based investigation of potential mechanisms of change

To identify robust, culturally sensitive mechanisms (processes) that might achieve change in the modifiable factors identified in our Ecocultural Model, we sought to understand in more detail the community assets that could be mobilised/invested/targeted towards change, and potential constraints or barriers. Our intention was to develop shared narratives that support local Somali refugees’ journeys from adversity to resilience, from isolation to connectedness, and from constraining to facilitating environments. Our local qualitative interviews (2012–2013) [3] were seen as stories providing ‘lessons for people who are planning change’ [62]. Using appreciative, situated inquiry [63,64], we investigated assets explicitly and systematically through qualitative case study (2020) [50], ethnographic observations (2017–18) [47], walkabouts with photography (2018–19) [13], and a further public involvement meeting exploring social networks and potential for social and environmental change (2019).

#### Public involvement and consultation to explore social networks and potential for social and environmental change

We met with a different set of local Somali community members (parents of children under 5 with Somali heritage, 10 participants, 2 hours, 2019) for discussion semi-structured around a social network tool adapted from Thomson et al [65]. We explored strengths, challenges, resources and desired outcomes from potential social change with 25 participants from a range of community, public, and voluntary sector backgrounds (4 hours), discussion semi-structured in world café format around the following themes: stories, songs and games; use of Somali language and culture; relationships, wellbeing, and trust; neighbourhood environments for play and interaction; and processes of inclusion/exclusion. We engaged local Somali children and young people in online workshops (10 participants aged 8–12, 6 hours, 2020), and also outdoor consultations (8 participants aged 10–14, 4 hours, 2021) to explore potential for local environmental change to improve opportunities for play and social interaction.

### Intervention design (2017-)

#### Co-production network for intervention design

To design the intervention presented, we have attempted to draw on locally and culturally congruent practices and networks [53,66–68]. We therefore held a further public involvement meeting with Somali community members (*n* = 10 (3 also participated in a previous meeting), 1 ½ hours, 2017), and have had ongoing discussions with associates of the Bristol Somali Resource Centre, Barton Hill Activity Club, and other Somali community members, and with statutory and voluntary sector staff, managers and commissioners.

**Figure 2.**
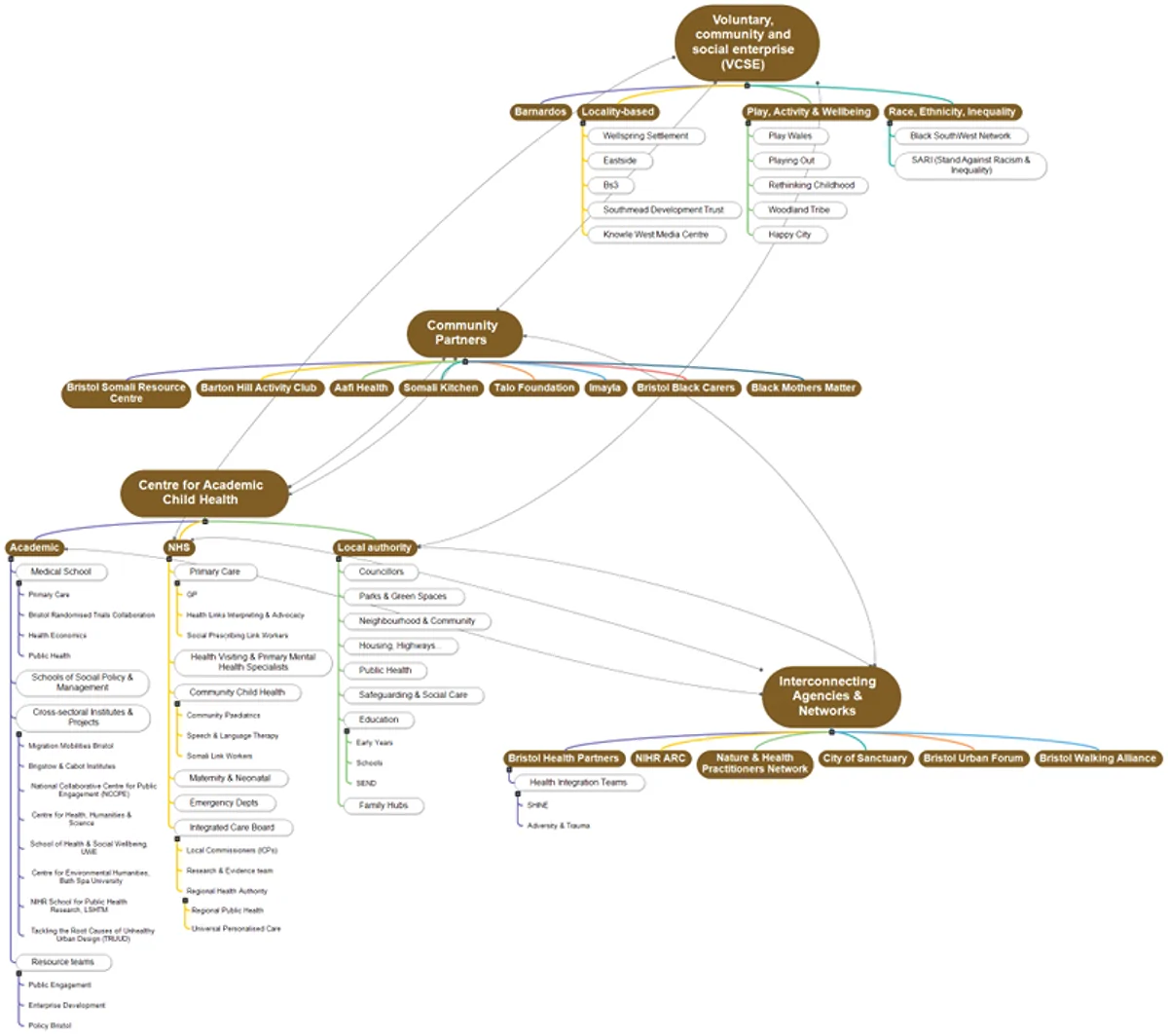
Co-production network for intervention development.

Looking beyond the local Somali community to wider networks relating to migrant heritage communities, we held a semi-structured focus group interview (1½h, 2023), and a series of co-production consultations with community group leaders/co-researchers (0.5–1.5 h, *n* = 6, 2024–25), to explore how intervention outcomes might be delivered in their specific community and neighbourhood context. Detailed refinement has followed as part of prototyping (see Figure 2) and co-production discussions as part of preparing funding applications.

A broad range of statutory, community/voluntary, academic and interdisciplinary organisations and networks have further contributed to the development of these approaches. Figure 2 (Co-production network for intervention development) depicts the network involved in the design of this intervention.

#### Mechanisms of change

Theorising the proposed intervention as an event in a complex system [69,70], we have attempted to describe the new settings and activities that it is hoped this intervention will facilitate, and how they might interact with the ecocultural context modelled above. Drawing on the Appreciative Inquiry-based investigations described above, we linked the relevant assets to each of the potentially modifiable factors from our Ecocultural Model, to describe how a peer support intervention in this context could bring about change. Our theoretical model for improving child development comes from Rogoff [71,72], who, after Vygotsky, argued that children learn through guided participation and joint problem-solving in shared, familiar cultural activities. This also links to Weisner’s ideas [73] about the value of sustainable daily activities & routine to foster wellbeing and provide a cultural environment for learning. Drawing on reviews and meta-analyses of successful interventions that support parents [74–93], we looked to include social support, co-parenting, parental sensitivity, and rewarding positive behaviour as key mediators, and mesh with local culture, language, parenting roles and wider contexts. We note successful interventions are often multi-faceted, build on existing resources/assets, model positive behaviour, target those at greater need (proportionate universalism), and work with groups. Key implications for this approach are that: a) culturally informed and attuned interventions will improve engagement and outcomes [53,54,87], b) successful interventions need to build on family strengths and address barriers [94,95], and c) a focus on social support and connectedness offers multi-faceted potential to improve outcomes [29,30].

Integrating the chosen mechanisms of change, we have specified the components of the proposed peer support intervention in order to deliver the desired effects. We present a linked logic model to summarise a multi-faceted peer support volunteer intervention and envisaged quantitative and qualitative outcomes.

### Prototyping

We refined our proposed intervention through a public involvement ‘taster day’ consultation (*n* = 25 families, 2017, during which Somali parents with children aged 0–2 could try out and give feedback on group activities proposed for inclusion in the intervention), through ongoing discussion amongst the co-producing intervention design team, and more recently through evaluations of peer support pilot projects (2024–25).

## Results

This paper draws together multiple steps in our collaborative, co-produced intervention development process – including evidence review, scoping of assets and needs, intervention design and prototyping.

### Evidence review and stakeholder consultation

The individual reports for the analysis of qualitative interviews [3], local population referral data [18], and systematic evidence reviews [48,49] are presented elsewhere. We also wished to review characteristics and components of effective interventions for children (including prenatally) and their families from migrant or refugee backgrounds; however, our searches of the same 12 databases used in the other systematic reviews found no evidence-based interventions addressing early childhood outcomes specifically for families in the Global North who have migrated from low‑income countries.

Key themes from each stakeholder consultation meeting were extracted and summarised and synthesised in the development of our Ecocultural model.

### Ecocultural model

**Figure 3.**
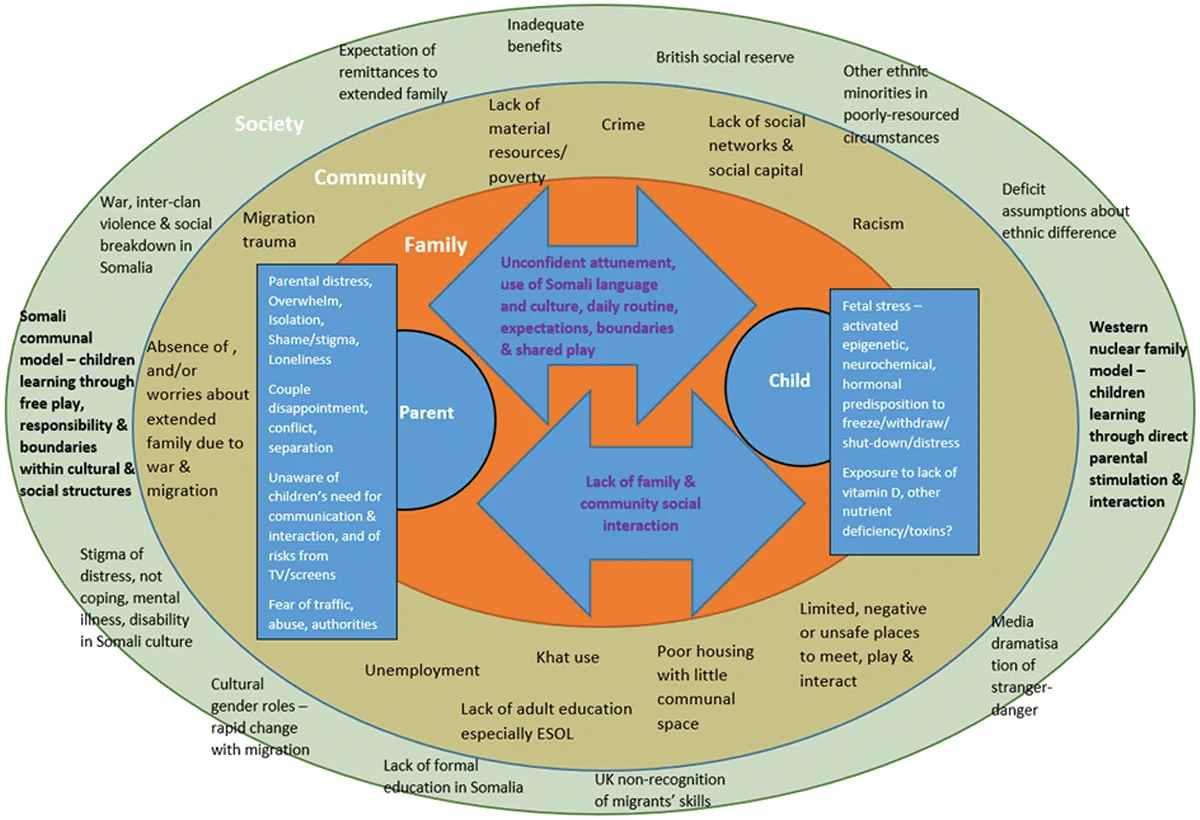
Ecocultural model of predisposing factors for early developmental problems in Somali migrants to the UK.

This model (Figure 3) describes a potentially syndemic process [96,97] whereby epidemics of maternal/parental lack of wellbeing and adverse early child development mutually reinforce amid multiple interacting sociocultural, economic, and migration vulnerabilities at family, community, and society levels [10,98–104].

Key mechanisms (acting at family level) are likely to include the epigenetic effects of stress, trauma, discrimination and social isolation [12,105–107], unconfident parent–child relationships [88,108], and lack of exposure to pro-developmental activities [25,109]. Negative experiences for parents can combine with lack of resources and difficulty negotiating the UK systems without extended family and community support to increase parents’ risk of stress, trauma, loss, relationship breakdown, stigma, and social isolation. Relevant stressors include: war, inter-clan violence, and social breakdown in Somalia, loss of extended family and community relationships through bereavement and migration, traumatic personal experiences, relationship conflict and separation, and a sense of shame/stigma about these life experiences. In the absence of extended family and community support that provided broad opportunities for play and social interaction in their country of origin, parents may not straightforwardly draw on the repertoires of 1:1 parent–child interaction that have become the norm within nuclear families in more individualistic societies. At a community level, lack of material, social, and environmental resources and a range of fear-inducing factors limit the opportunities for the social connections that could buffer families’ experience of stress. Wider societal factors act in a variety of marginalising ways to interfere with families’ sustainability in terms of financial (inadequate benefits, expectation of sending remittances to family abroad), social (deficit assumptions about ethnic difference, non-recognition of migrants’ skills, authorities’ decisions about housing allocation setting up competition with other ethnic minorities in poorly resourced circumstances) and cultural (rapid change in gender roles such as housing tenure; fears due to media dramatisation of stranger-danger in an unfamiliar cultural environment) resources, contributing to stress and social isolation. These multi-layered and mutually potentiating challenges serve to contribute to impaired early child communication and social-emotional development.

Importantly, these problems occur at the meeting point (through family migration) of two very different systems of child-rearing: a communal African/Somali model of children learning through free play, responsibility and boundaries within cultural and social structures contrasting with a Western nuclear family model of children learning through direct parental stimulation and instruction. Somali parents in the UK may have had positive childhood experiences of their own that they are not able to draw on due to the very different physical and social context in the UK or may have had disruptive and traumatic childhood experiences due to war and migration. Additionally, trauma (child or adult) may disrupt the ability to reflect (metacognitively) on the emotional effect on relationships in a way that adds to the difficulty of providing responsive current parenting [110].

We also hypothesise that children born after stressed pregnancies (https://developingchild.harvard.edu/resources/infographics/what-is-epigenetics-and-how-does-it-relate-to-child-development/) may be born in a state more likely to react strongly, or ‘shut down’ at difficult or distressing moments, which in turn may potentiate parental stress (through tiring and unrewarding parent–hild relationships), and social isolation (due to children’s atypical and stigmatised behaviour) which contribute to lack of critical early opportunities for play and social interaction. While screens (TV, computers and other devices) may focus children’s attention and keep them quiet, they are not coordinated communication partners for pre-verbal children; prolonged and solo use risks delaying the development of communication, learning and emotional regulation skills [111]. At the centre of this description therefore lies a mutually reinforcing triad of parental stress, family isolation and child developmental difficulties.

Potentially modifiable factors in this model include: parental stress, and consequently children’s epigenetic, relational and social environments; the social (people, settings and activities) and physical (housing, communal space and outside space) environment within which families meet, parents receive support and children play; and activities parents prioritise and de-prioritise for their children.

### Asset-based investigation of potential mechanisms of change

#### Case study, ethnographic observations, and walkabouts with photography

Our explicit and systematic exploration of assets with potential to support change has included a case study pointing to the potential of community activities and organisations to foster positive social networks and peer support [50], a description arising from ethnographic study of distinct and complementary benefits of structured and unstructured activities [47], and mechanisms to develop shared visions for local environmental improvements and communicate them successfully to local decision-makers [13].

#### Social networks; social and environmental change – public involvement and consultation

In consultations about social networks, participants described widely-varying breadth and depth of social networks, for themselves and others, at times of having young children, depending on the social, economic and physical resources available to them, and in particular their sense of connectedness with extended family, local communities (Somali and wider) and organisations/services, and of the importance of this for wellbeing, as well as for children’s opportunities for play and interaction that could support their developmental progress. Consultations about social change identified a wide range of assets and barriers for inclusion in the intervention design process.

### Intervention design

#### Mechanisms of change

Table 1 sets out the putative mechanisms of change, linking each potentially modifiable factor with relevant Somali community assets to mobilise or target, what a peer support intervention could provide, and their intended effects. The focus groups and co-production consultations with community leaders and stakeholders pointed especially to the potential value, through culturally coordinated action, of activating latent assets (assets present in families’ and communities’ heritage and experience, but currently hidden, suppressed or silenced).

**Table 1.** Potential mechanisms of change.

**Potentially modifiable factors**	**Relevant Somali community assets to mobilise/target**	**Peer support intervention providing:**	**Intended effect**
Parental stress, and consequently children’s epigenetic, relational and social environments	-Families’ community support network -Shared responsibilities and duties (including child care shared), and local support when facing difficulties	●Relationships and emotional support ●Modelling, education, motivation, feedback and opportunities for discussion with peers ●Practical support/advocacy	●Improved parent wellbeing and reduced stress, with buffering [112] of negative experiences ●Parents experiencing themselves as having useful knowledge and practice to offer their children ●Reduced valency of shame/stigma, less limiting of choices about involvement in social activities ●Easier negotiation of UK systems and services
The social (people, settings and activities) and physical (housing, communal space and outside space) environment within which families meet, parents receive support and children play	-Extended families and communities coming together for shared, culturally relevant activities (stories, songs, proverbs) -Familiar and trusted neighbours taking communal responsibility for children’s safety -Children’s free play outdoors, socialising together, using their surroundings to play and learn creatively, actively and safely	●Active outreach and engagement, with inclusive philosophy ●Group activities providing social involvement directly ●Supportive social context as nucleus ●Practical support/advocacy - for environmental improvements	●Greater community social connectedness and inclusion, with sense of belonging, pride and inspiration for future generations ●More mutually supportive conversations, interactions and activities within Somali community and with wider communities ●Increased participation by isolated families, in a wider range of settings and activities, and with more supportive relationships within the local community ●Parents able to access child care through social networks ●Improved local environments; reappraisal of safety concerns; more opportunities for free play
Activities parents prioritise and de-prioritise for their children	-Children in Somalia given expectations, opportunity, encouragement, guidance and responsibility from an early age to develop a range of skills which support their confidence, independence, maturity, and ability to communicate and socially connect within a shared cultural framework	●Modelling, education, motivation, feedback and opportunities for discussion with peers ●Setting providing high quality, open-ended play opportunities for children to explore and interact in, and parents to gain confidence and experience ●Public health messages about pro-developmental activities & the risks from excess screen access	●Confident, positively-focussed parent-child relationships, successfully problem-solving and repairing relationships at difficult moments (demand, confusion, distress) ●Parents providing children with sustainable daily activities and routine ●Parents reclaiming and transforming culturally-meaningful pro-developmental activities from Somali heritage into a UK context ●Parents negotiating more pro-developmental activities (e.g. interactive play; positive communication…) and less time in anti-developmental activities (e.g. screen time; close attention to negative behaviours/distress) ●Children playing and interacting positively, creatively ●Children exposed to/guided in pro-developmental activities

#### Intervention components

The intervention is intended to be tailored flexibly to its local context and adjusted according to feedback, to facilitate cultural relevance, collaboration, uptake, and impact [31,32,113–116].

To incorporate the relevant assets in a peer support intervention in this context, bringing families together and enabling them to meet their needs, our proposed intervention therefore has three components:

1) Proactively engaging, enabling, and advocating for families.

Family engagement, enablement, and advocacy are seen as a foundation for regular group attendance and positive experience of the intervention overall. Peer supporters offer group or 1:1 consultations during unstructured group time, or by arrangement in other settings (community or home). These are designed to identify and mobilise family assets, needs, and social networks (existing and potential), meet their needs through individual or collective action, and support access to other local opportunities. Prepared materials provide clear public health messages about pro-developmental activities and the risks from excess screen use.

2) Organising group activities.

Weekly group activity sessions provide informal, play‑based sessions giving parents and pre-school children opportunities for shared, safe, inclusive and non-judgemental play and learning. Training, and collaboration with experienced early years practitioners ensures the activities and toys/equipment involved are developmentally of high quality – ie open-ended, responsive, stimulating and challenging [117]. Providing refreshments increases the opportunity for participants to experience social support and contribute to informal opportunities for engagement, enablement, and advocacy (see above) where needed.

External facilitators can be invited for additional structured sessions, programmes, and connections. Pilot work seeking to address specific key mechanisms has identified the following as potentially valuable: sharing Somali stories and games; addressing principles of communicating with babies and children through group activities; supporting child and parent attachment, emotional wellbeing and communication, and offering embodied trauma-aware activities and relaxation.

3) Advocacy for neighbourhood environmental improvement.

Regular facilitated meetings seek to identify and advocate for improvements to local environments for play and social interaction, empowering participants through negotiating change with neighbourhood and Local Authority processes and resources.

#### Logic model

**Figure 4.**
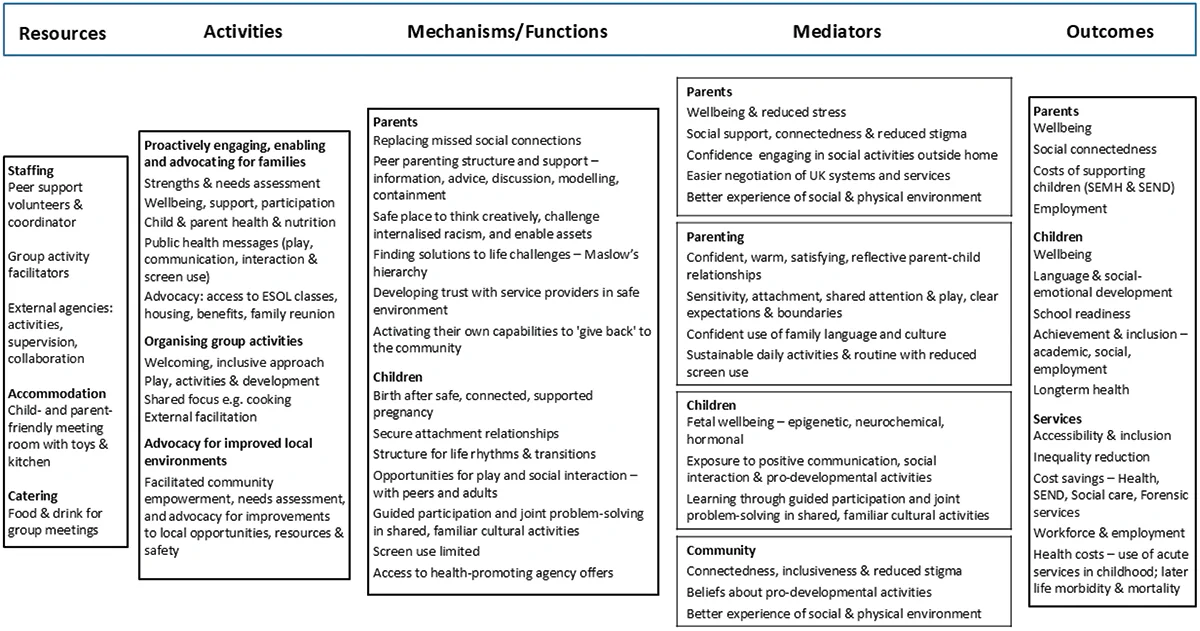
Logic model for Find village peer support intervention.

The logic model for our proposed intervention summarising a multi-faceted peer support volunteer intervention and envisaged quantitative and qualitative outcomes is presented in Figure 4.

### Prototyping

#### Public involvement taster day

This day was attended by 25 Somali parents with children aged 0–2, during which families could try out possible externally facilitated group activities proposed for inclusion in the intervention and give feedback about their acceptability and perceived usefulness. All components of the intervention trialled received positive feedback. Activities appeared to help participants make experiential connections between child- and parent- wellbeing, and communal activities, and to establish a firm basis for further engagement.

#### Social prescribing pilot

We used observations and informal interviews to explore potential mechanisms of change in a Somali-led pilot green social prescribing intervention [45,51]. We observed local and cultural knowledge appearing to permit confident, proactive outreach which engaged isolated community members in positive social connection. An inclusive, non-judgmental, reflective, and solution-focused approach appeared to help develop self-care skills and destigmatise discussion of wellbeing/mental health. Nature-based activities helped participants see their natural environment as accessible and part of their lives, widening marginalised community members’ ‘comfort zones’ in the world around them. These benefits were hypothesised to interlink as a potentially sustainable cycle.

#### ‘First 1001 days’ peer support pilots

##### Peer support pilot 1: somali-led

Our partner Barton Hill Activity Club (BHAC) delivers a Somali-led pilot intervention in the Barton Hill area of Bristol, aiming to reach out to all families with migrant heritage where there are pregnancies or pre-nursery age infants in a defined local area (the reach area of the local Nursery/Children’s Centre). Taking a proportionate universalist approach, the intervention is open to all, while prioritising the attendance of stressed and isolated migrant parents otherwise out of contact with community and services, through engagement, advocacy and support activities.

Components: (1) Local parents trained to identify their own and community strengths to create parent–child activity and support groups; (2) Peer supporters networked with local community and linking with local services; (3) Safe spaces for play and interaction; (4) Culturally coordinated activities; (5) Accessibility for all members of the family; (6) Proportionate universalist approach.

Through Find Your Village peer support groups, organisers/peer supporters report that participants ‘start to relate to agencies through a cultural context that is safe and familiar.’ Partner organisation Barnardos’ evaluation report of these groups is accessible here: https://research-information.bris.ac.uk/ws/portalfiles/portal/470985284/FYV_report_Sep_25.pdf

##### Peer support model 2: African diaspora maternity-centred

Our partner Black Mothers Matter (www.blackmothersmatter.org) supports and celebrates Black pregnancies, working to improve information, support, services, and research for Black women and their babies. Supported by local NHS commissioners as part of a Maternity Champions pilot to improve experience and outcomes for Black women and their babies and additional charitable funding, they offer a multi-modal programme with the following components: (1) Antenatal programme by and for Black women to complement existing NHS antenatal courses and prepare participants for experiences specific to Black people; (2) Maternity champions inreach to local NHS services; (3) Ongoing peer support and shared activities from pregnancy until children start nursery.

Organisers/peer supporters say ‘Communal support around childrearing is part of the shared history of black communities. Bringing people together who share part of their identity or history allows them to focus holistically on the things going on in their lives … Being able to recreate that feels really comforting.’ Our evaluation of these groups is reported here https://research-information.bris.ac.uk/en/projects/black-mothers-matter-community-groups-evaluation, involving observations, interviews, and survey data.

## Discussion

### Summary

This study presents an innovative, theory-driven approach to developing asset-based community peer support interventions for wellbeing and early child development, drawing on Bristol (UK) exemplars, and designed for transferability to other contexts of migration and marginalisation. The key functions of this asset-based peer-support intervention are: 1) proactively engaging, enabling and advocating for families; 2) organising group activities; and 3) advocating for neighbourhood environment improvements. Anticipated benefits include parent wellbeing; parent–child relationships and parenting confidence; child wellbeing and development; community social networks; and relationships with local agencies.

### Transferability: focus on functions and assets

The focus on functions is intended to facilitate local communities, organisations or partnerships to co-design their own adaptations and solutions to meet local needs [118]. In our two pilot exemplars, local people decided how these ideas should be put in practice to work for their communities. Following Hawe et al [69], the impact of an intervention depends on its relationship with its context, within complex social and environmental systems [119]. This intervention aims to mobilise the assets of communities with migrant heritage, including shared cultural understanding and communal, collective child-rearing practices, in order to provide effective mutual support. It looks for the language, relationships, roles, and activities of participants and other community members to evolve in positive ways, incorporating the aims of the intervention, so that change is sustainable. Positioning the intervention at the meeting point of communal/collective and nuclear-family/direct stimulation approaches to child-rearing, the intervention seeks to draw on the strengths of both through attention to relational and participatory opportunities [120], creating places where parents and children can interact to benefit confidence, wellbeing and development [121]. The approach can be seen as aligned with attention to social determinants of health [122], noting recent attention to the importance of trust in reducing health inequalities [41,123], and with the United Nations sustainable development goals (https://sdgs.un.org/goals).

### Mixed methods evaluation and implementation

Looking towards implementing these approaches on a wider scale, it will be important to consider, and therefore evidence richly, in context, the skills and relationships necessary for effective engagement, fostering trust, supporting change, and addressing potential barriers at individual, community, and agency levels [124]. Mixed methods evaluation should therefore address its potential ‘to gain traction within its system’, looking at the fidelity of implementing an intervention’s functions rather than components, as part of evaluating implementation, refining the understanding of mechanisms of change, and the interrelationship with complex, intersectional contexts [114,115,125]. We consider parent wellbeing, social support/connectedness, and confidence with childrearing, as highly desirable in their own right, and also likely to play important mediating roles in childrens’ outcomes [126]. We have identified and piloted the acceptability of key measures to provide early indication of benefit (evaluating parent wellbeing, parenting self-efficacy, social support and capability), that can also contribute to economic costings. There is also potential for multi-dimensional, cumulative benefit over substantial timeframes which can be evaluated [127]. We would also explore potential to link within-project data about attendance, wellbeing, and early child development with health economic modelling about costs to services and society of relevant adverse outcomes [128]. At the same time, the experience of being evaluated for individual participants, and the requirement to demonstrate efficacy for organisations offering peer support interventions, presents obligations which may challenge the trust required to work effectively with marginalised community members. These considerations will, flexibly and iteratively, require continuing change in the intervention design framework and process [42]. We therefore emphasize the need to develop, evaluate, and implement interventions within a collaborative, asset-based, co-design process aiming to provide value to communities and agencies [124].

### Reach and relevance

In 2023, 37.3% of live births (220,470) were to parents where either one or both were born outside the UK, increasing from 35.8% in 2022; 23.8% of live births (140,852) were to parents where either one or both were born outside the ‘Global North’, increasing from 22% in 2022 [2]. Experience and outcomes for ethnically marginalised women in pregnancy and childbirth, and for their children’s health, wellbeing, development and life chances face multiple, interconnected, systemic challenges. We therefore think these approaches have potential for broad reach and relevance. Many of the processes and functions we describe may also be relevant for families and communities in other situations of disadvantage and marginalisation, including white urban and rural underserved or isolated communities.

### Implications for practice, commissioning and research

This flexible approach to co-produced asset-based peer support in the first 1001 days of life for communities with migrant heritage has potential to be delivered on a large scale, adapting to local strengths and needs, aiming to reduce current inequalities in experience and outcome for children and families, and costs for families and services. We hope the flexibility of this approach will enable other local communities to develop equivalent peer support interventions for families with migration heritage. The approach’s sustainability on a local level can therefore arise from activating communities’ assets and increasing social connections, while also enabling better relationships and with agencies.

This approach also has potential to inform wider policy and commissioning [129]. The intention is that the learning from this work can help tailor statutory policy and practice in health and local authority services for migrant families and communities. This includes early years, trauma-informed, and social prescribing practice, and the range of services and planning processes that affect neighbourhood environments. It may assist the design of neighbourhood preventive services to improve health and wellbeing, and reduce inequalities. We believe this has potential to be a highly cost-effective initiative when set against the very substantial longer term multi-sectoral expenditure on young people with negative educational, employment and offending outcomes, and later health costs associated with social disadvantage and social isolation [122], and hope to model the potential economic benefits within routinely collected, linked datasets. Resourcing these kind of interventions at scale will require attention to the wider systems within which they may be commissioned [52,130,131].

### Limitations and strengths

Limitations include the lack of formal, mixed-method and economic evaluation of specific interventions. We also acknowledge: each of the multiple data collection approaches has its own limitations; the imperfection of attempts to consider multiple perspectives and equalise power during co-production processes within multi-layered contexts of structural inequality; and the intervention design having taken place primarily in one urban centre of the UK. Strengths of this report include the 10 ‑year collaboration and co-production relationships in asset-based intervention design, multiple triangulating methodologies, and the flexible approach to support transferability. While we are aware of adaptable parenting interventions such as Ladnaan [132] and SPARE [133], and community-centred approaches such as NEON [134], to our knowledge this is the first initiative that offers a co-design approach to community peer support focused on the first 1001 days for migrant heritage communities in the Global North.

We do not anticipate significant ethical challenges from this intervention, with potential risks for participants and society considered low. Aiming to ensure the social support offered is beneficial to all, peer support activities have been designed to be supportive, inclusive, practical, and enjoyable, with the inclusive approach supported by regular reflective team meetings. It will nonetheless be important to monitor the accessibility and inclusiveness of any such intervention within the delivering team and as part of evaluation [135,136].

## Conclusion

This study presents an innovative, theory-driven approach to developing asset-based community peer support interventions for wellbeing and early child development, drawing on Bristol (UK) exemplars, and designed for transferability to other contexts of migration and marginalisation. Key functions of this asset-based peer-support intervention are as follows: (1) proactively engaging, enabling, and advocating for families; (2) organising group activities; and (3) advocating for neighbourhood environment improvements. We recommend mixed methods evaluation of effectiveness and implementation within a collaborative, asset-based, co-design process, aiming to provide value to communities and agencies. We envisage this facilitating local communities, organisations or partnerships to co-design their own adaptations and solutions to meet local needs according to these principles, at scale, in interrelationship with complex, intersectional contexts.

## Supplementary Material

TIDieRPHP reporting guidelines self appraisal.docx

## Data Availability

There is no specific dataset linked to this article but further information can be made available by the corresponding author on reasonable request.
