# Supplementary material for: Find your village: co-designing a community asset-based peer support intervention for wellbeing, social connectedness, and early child development among migrant families during the first 1001 days
Source: Glob Health Action. 2026 Sep 21;19(1):2731693. doi: 10.1080/16549716.2026.2731693 (PMC13600321; doi:10.1080/16549716.2026.2731693)
Supplement: Supplementary file 1 [file ZGHA_A_2731693_SM4727.docx]

**TIDieR-PHP reporting guidelines self-appraisal** (Campbell M, et al. TIDieR-PHP: a reporting guideline for population health and policy interventions. Bmj. 2018;361. <https://doi.org/10.1136/bmj.k1079>.)

| **Item** | **Comment** |
| --- | --- |
| 1. Name: Find Your Village flexible community asset-based peer support intervention to improve wellbeing, social connectedness and early child development in the first 1001 days for communities with migrant heritage | Included |
| 2. Why: Describe the logic, mechanisms, or rationale of the intervention, clearly linking intervention elements to the expected effects on immediate or longer term outcomes (or both). | Clear theory and rationale articulated |
| 3. What materials: Describe any materials used in the intervention | Co-development of interventions in individual pilot projects, using oral presentation of intervention development theory, findings and recommendations, and collaborative, iterative process. |
| 4. What and how —Describe how the intervention was planned, established, and intended to be delivered. | Detailed description included. |
| 5. Who provided: Describe the provider of the intervention, including legal status and powers, field organisations and staff responsible for planning, implementation, monitoring and enforcement. | Flexible, not mandated. |
| 6. Describe the type of location (eg, school, community centre) and the geographical scope of the intervention (eg, national, regional, city-wide). Where relevant, describe the historical, cultural, socioeconomic, or political background to the intervention. | Location: Community-based, flexible, local neighbourhood scope |
| 7. When and how often: Describe when the intervention was implemented, how long it remained in place, and, if applicable, the number, duration, and scheduling of occasions. | Pilot interventions currently ongoing (5 and 1.5 years respectively); group sessions weekly with peer support volunteer outreach in between groups |
| 8. Planned variation: Describe and provide the reason for any variation or tailoring that was planned or allowed for in the design of the intervention.   Unplanned variation: Describe and provide the reason for any unplanned variation or modifications in the intervention that were made after the intervention commenced. | Flexible intervention for local co-production to design approaches to achieving the core functions described.  Interventions flexible for local adaptation and refinement to achieve core functions. |
| 9. How well: Describe any strategies used or actions taken to maintain fidelity of the intervention (ie, to ensure that the intervention was delivered as intended).   Describe the fidelity of the intervention (ie, the extent to which the intervention was delivered as intended). | Continuing collaborative discussion and monitoring by the co-producing team.  Fidelity to core functions (rather than components) described in qualitative evaluations. |
